# Supplementary material for: Self-reported medication adherence among patients with diabetes or hypertension, Médecins Sans Frontières Shatila refugee camp, Beirut, Lebanon: A mixed-methods study
Source: PLoS One. 2021 May 10;16(5):e0251316. doi: 10.1371/journal.pone.0251316 (PMC8109801; doi:10.1371/journal.pone.0251316)

**S1 File. Quantitative component questionnaire including the MMAS-8 - English and Arabic versions**

| Date of birth: ____/____/________ | | ***English version***  Study ID #: _______________ | | | |
| --- | --- | --- | --- | --- | --- |
| Gender: 🞎Male 🞎 Female  **If patient is between 7 & 18y.o. verbal assent obtained 🞎Yes 🞎No** | | | | | |
| 🞎 Other; *Time since diagnosis*: _________ | | 🞎 Hypertension | 🞎 Diabetes | | Diagnosis: |
|  | |  |  | | |
|  | | 🞎 No | 🞎 Yes | | Employed: |
|  | 🞎 Lebanese | 🞎 Palestinian | 🞎 Syrian | | Nationality: |
| 🞎 Palestinian Lebanese | | 🞎 Palestinian Syrian | | |  |
| 🞎 Not registered | | 🞎 Registered | | Registration status: | |
| Literacy: 🞎Reads and writes 🞎Reads only 🞎Illiterate | | | | | |
| ______________________ | | | | Area of residence: | |
| _____ Months _____Years | | | | Duration of displacement: | |
|  | | Number of chronic medication: __________ | | | |
| Attended PSEC: 🞎 No 🞎 Yes, *how many sessions on the program so far*: ______  Latest HbA1C measurement: _______________ Blood pressure: _______________  Date of latest HbA1C: _____________________ Date of latest Blood Pressure: ________ | | | | | |
| **Morisky medication adherence scale** **(MMAS-8):** | | | | | |
| 1. Do you sometimes forget to take your medicine? | | | | | |
| 🞎 Yes 🞎 No | | | | | |
| 1. People sometimes miss taking their medicines for reasons other than forgetting. Thinking over the past 2 weeks, were there any days when you did not take your medicine? | | | | | |
| 🞎 Yes 🞎 No | | | | | |
| 1. Have you ever cut back or stopped taking your medicine without telling your doctor because you felt worse when you took it? | | | | | |
| 🞎 Yes 🞎 No | | | | | |
| 1. When you travel or leave home, do you sometimes forget to bring along your medicine? | | | | | |
| 🞎 Yes 🞎 No | | | | | |
| 1. Did you take all your medicines yesterday? | | | | | |
| 🞎 Yes 🞎 No | | | | | |
| 1. When you feel like your symptoms are under control, do you sometimes stop taking your medicines? | | | | | |
| 🞎 Yes 🞎 No | | | | | |
| 1. Do you ever feel hassled about sticking to your treatment plan? | | | | | |
| 🞎 Yes 🞎 No | | | | | |
| 1. How often do you have difficulty remembering to take all your medicine? | | | | | |
| 🞎 Never 🞎 Once in a while 🞎 Sometimes 🞎 Usually 🞎 All the time | | | | | |

***Arabic version***


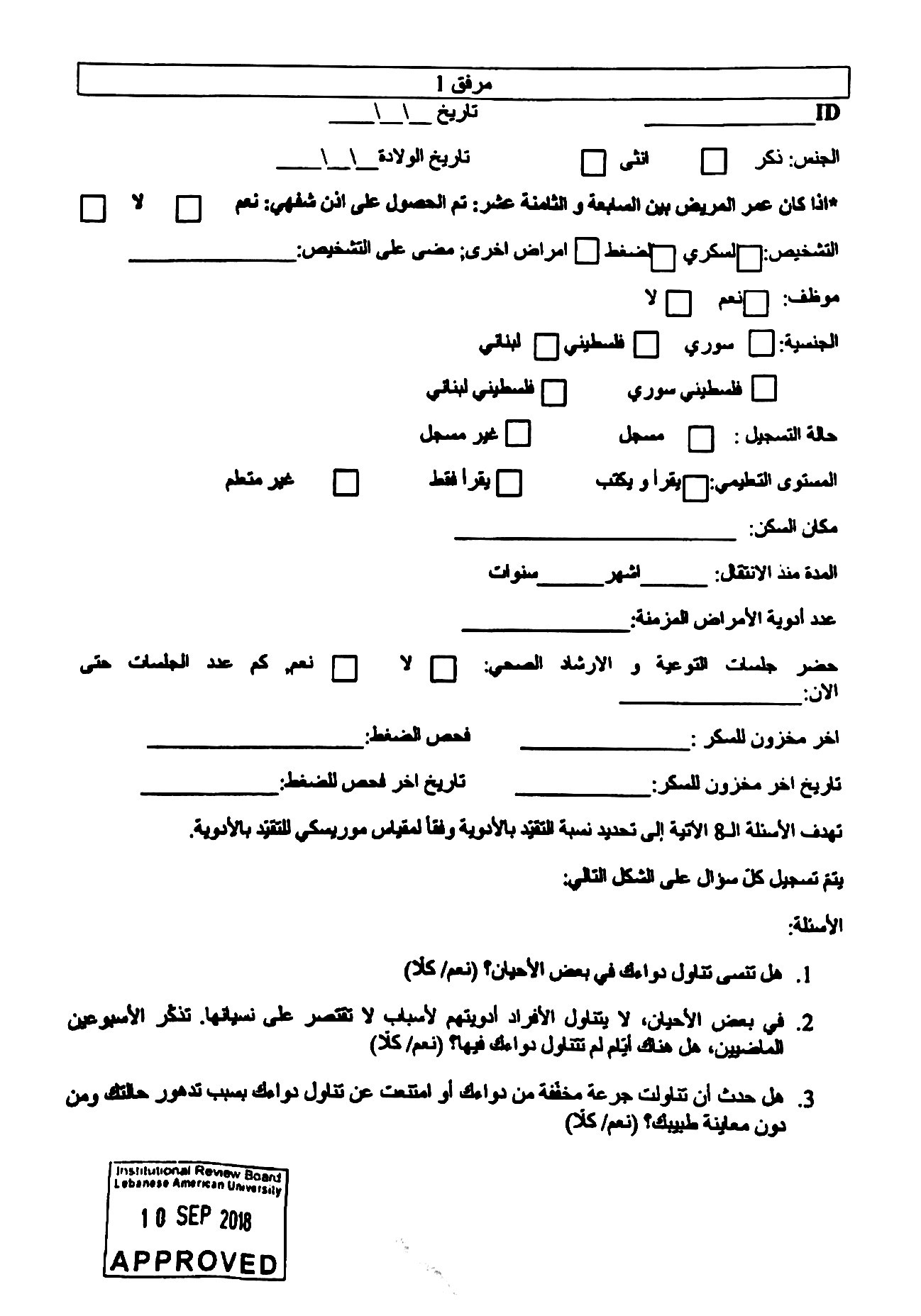


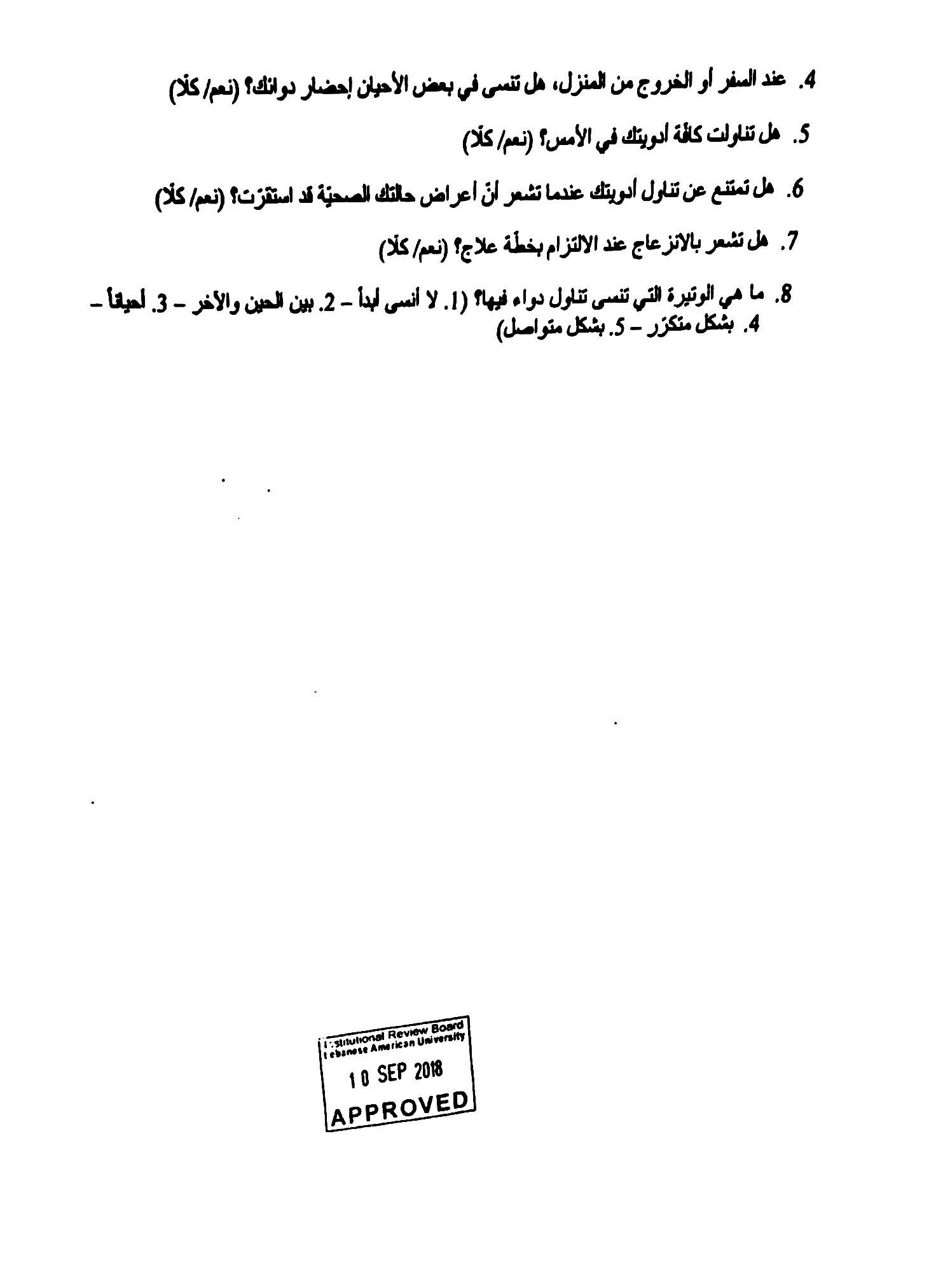

Supplement: S1 File — (DOCX) [file pone.0251316.s001.docx]
