# Supplementary material for: Self-reported medication adherence among patients with diabetes or hypertension, Médecins Sans Frontières Shatila refugee camp, Beirut, Lebanon: A mixed-methods study
Source: PLoS One. 2021 May 10;16(5):e0251316. doi: 10.1371/journal.pone.0251316 (PMC8109801; doi:10.1371/journal.pone.0251316)

**S2 File.** **Qualitative interview guide for patients – English and Arabic versions**

***English version***

**NCD Diagnosis:** 🞎 Diabetes 🞎 Hypertension 🞎 Other

**Date of Interview: Interview start / end time:**

**If minor, verbal assent obtained: □ Yes □ No**

After a brief introduction to the participant regarding the purpose of the interview, the interviewer will obtain informed written the consent for the interview and for an audio recording.

1. Can you tell me a bit about yourself and the the chronic disease problems do you/your child currently have/has that are being managed by the MSF clinic? [Probe: does the patient know what their current diagnosis, how well they know it, and why they are taking medication]
2. What medication(s) are you/your child taking at this time and for which diagnoses? [Probe: that they understand each medication by name and for which diagnosis it has been prescribed]
3. What frequency are you/your child supposed to take each medication and what time of day? [probe: understanding of the medication(s) prescribed and how frequently they are to be taken]
4. Do you think it is important to take/give your medications every day/as prescribed? Why is this important -or not important- in your opinion?
5. It is sometimes challenging to take/give medications as prescribed. How much has this been challenging for you? What are the challenges you face? [Probe: personal motivation (sometimes tired of taking the medications, number of medications taken together), being busy with a number of other priorities]
6. Everyone tends to miss their medication dose sometimes for different reasons, what makes you miss yours sometimes? Is it easy to miss doses? [Probe: social obligations (work, children, etc.), cultural factors (fasting) , I rely on my husband/parent and he is sometimes out/he forgets, when I feel fine I allow myself not to take them]
7. Do you consider yourself as someone adherent to his medications? Why do you think you have/not have been able to be properly adherent? [Probe: because of time of day needs to be taken, frequency (once versus twice a day), travel, work]
8. Do you ever run out of medications before you get a refill? [Probe: how often this is occurring] What are the reasons you run out of medication? [Probe: can’t get to the clinic because of work, cost to travel to clinic, checkpoints, other causes]
9. How do you think you can improve your medication adherence? What would help you improving it? What can the organization (MSF) do in order to help you in that? [Probe: better understanding the disease, more knowledge about medications, organizing my pills, getting reminders, more support from family/friends]

**For children:**

1. Was your child explained his/her diagnosis? [Probe: did a doctor or nurse explain the diagnosis or medication to the child?]
2. Do you face any challenges with your child for his/her medication intake [Probe: child is scared from needles (for DM1), child resistant to take medication on daily basis]

The interviewer will complete the interview by acknowledging the time spared by the participant from his/her busy schedule. They will also give a summary of the notes taken and confirm the same from the participant.

***Arabic version***


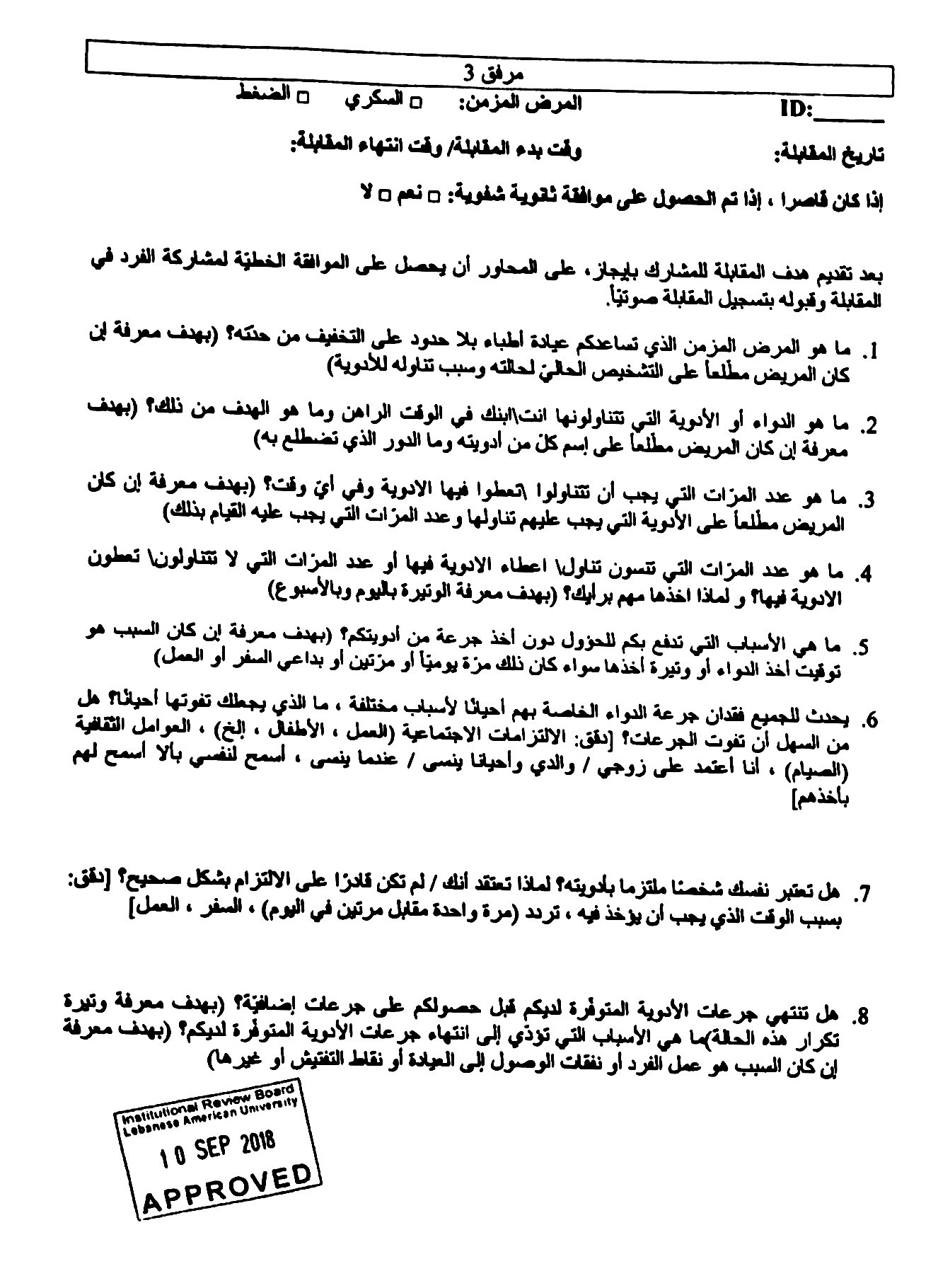


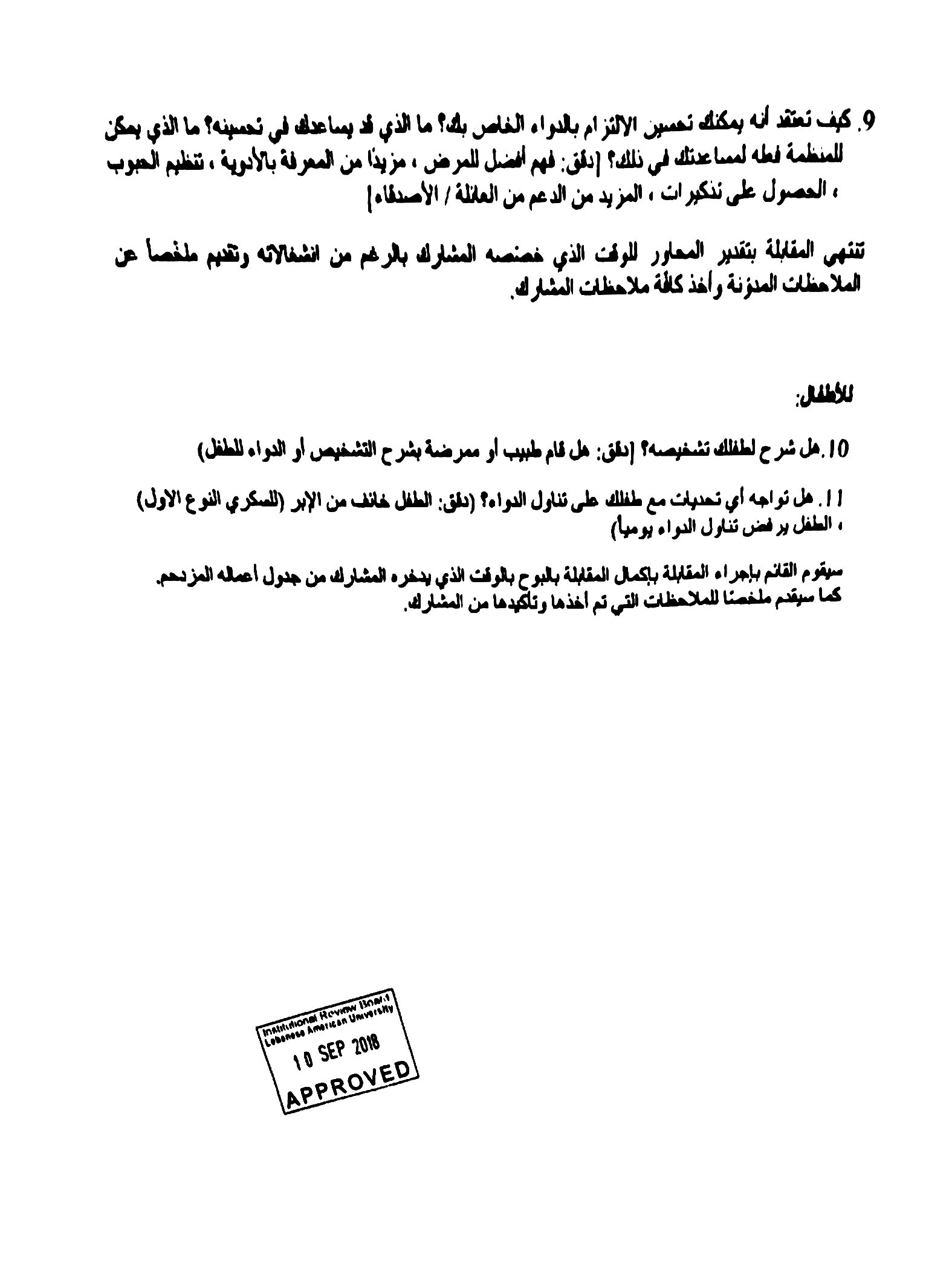

Supplement: S2 File — (DOCX) [file pone.0251316.s002.docx]
