# Supplementary material for: Self-reported medication adherence among patients with diabetes or hypertension, Médecins Sans Frontières Shatila refugee camp, Beirut, Lebanon: A mixed-methods study
Source: PLoS One. 2021 May 10;16(5):e0251316. doi: 10.1371/journal.pone.0251316 (PMC8109801; doi:10.1371/journal.pone.0251316)
Supplement: Attachment — Submitted filename: PONE-D-20-36223 Reviewer Comments.docx [file pone.0251316.s003.docx]

This is an important and well executed study that should be published. The work contributes to a sparse evidence base on an important and timely topic. The article is well written with mostly minor suggestions for improvement:

1. Introduction (line 84): Clarify that the 50% low adherence estimate is global not population-specific
2. Introduction (lines 93-94): There is also a relatively new (2020) article on exactly this: <https://doi.org/10.1007/s40200-020-00638-6>
3. Methods (line 130): For those on insulin and oral medication, is “adherence” to one or both?
4. Methods (“MSF model of care” & generally throughout): To what extent do MSF patients receive care or medication outside MSF clinic(s)? This may not be known but is a question that came up for me throughout, so may be worth addressing, if possible, along with implications.
5. Methods (line 217): Suggest mentioning in the limitations section potential limitation regarding gender issues since a female conducted all interviews and possible gratuity bias since research was done by known providers/at clinic.
6. General comment on analysis and presentation of results: It is complicated to lump insulin with oral Rx adherence; the issues and drivers of adherence are often different. I would suggest exploring analysis separately for insulin vs oral and including as supplementary table(s). At a minimum, the potential influence of analyzing these together should be mentioned.
7. Results (line 308): How many of those on >1 medication were on insulin + oral?
8. Results (“Multivariate Analysis” section): The absence of significant predictors may also have been related to distribution of predictors/adherence since the sample size is not so large, limiting the ability to detect weaker associations.
9. Results (lines 352-3): It is unclear if this is from the current study findings or previous studies/assumptions
10. Discussion (lines 499-502): Wording in this sentence is confusing
11. Strengths & Limitations (lines 518-20): True but removing one of the well-known drivers of low adherence (i.e., cost) may also be a limitation
12. Suggest mentioning limiting the sample to MSF patients as a limitation in terms of representativeness; those not receiving care from MSF may have different adherence prevalence and predictors
13. Conclusion (lines 537-8): Does this sentence draw directly from the study findings? This claim’s basis would require external reference(s), no?
